# Application of Spectral Approach Combined with U-NETs for Quantitative Microwave Breast Imaging

**DOI:** 10.3390/s25082450

**Published:** 2025-04-13

**Authors:** Ambroise Diès, Hélène Roussel, Nadine Joachimowicz

**Affiliations:** 1 Sorbonne Université, CNRS, Laboratoire de Génie Electrique et Electronique de Paris, 75252 Paris, France; ambroise.dies@sorbonne-universite.fr (A.D.); helene.roussel@sorbonne-universite.fr (H.R.); 2 Université Paris Cité, F-75006 Paris, France

**Keywords:** microwave imaging, U-NET, deep learning, backpropagation, spectral techniques, anthropomorphic breast model

## Abstract

This study focuses on breast imaging. A spectral approach based on the Fourier diffraction theorem is combined with a pair of U-NETs to perform real-time quantitative human breast imaging. The U-NET pair is trained based on the input of an induced current spectrum and the output of a contrast dielectric spectrum. A spectral database is constructed using combinations of anthropomorphic cavities. The weighted mean absolute percentage error (WMAPE) loss is associated with the Adam optimizer to perform optimization. Numerical results are presented to validate the proposed concept to demonstrate the transformation brought about by the U-NETs.

## 1. Introduction

In inverse scattering for microwave breast imaging, a quantitative description of the breast, corresponding to its dielectric properties, is calculated from the measurement of the scattered field induced by the presence of the breast. From a mathematical viewpoint, this is an ill-posed problem, due in part to the lack of access to evanescent waves, and a non-linear problem because of the multiple scattering effects inside the breast. To solve this issue, iterative optimization methods incorporating prior information have been developed to minimize the deviation between the calculated and measured scattered field distributions. The contrast source inversion method (CSI) [1,2,3], the distorted born iterative method (DBIM) [4,5], Gauss–Newton (GN) [6], and the Broyden–Fletcher–Goldfarb–Shanno method (L-BFGS) [7] are examples of deterministic optimization algorithms used for medical applications. Recent developments in deep learning methods, such as perceptrons (ANNs) and convolutional neural networks (CNNs), have been under investigation. These are deterministic methods based on a stochastic optimization process that incorporates an entire database as prior information. Numerous studies have shown that it is possible to improve microwave imaging techniques by combining them with deep learning, as proposed in [8,9,10,11].

More specifically, Yingying Qin et al. [2] and Mojabi et al. [12] investigated the use of multichannel CNNs and U-NETs, respectively, to combine microwave and ultrasound images using the databases described in [13] and [14]. In [15], Ambrosanio et al. used smart-automated generation of 2D maps to create a large database on which they trained an (ANN) to transform the measured scattered field into dielectric properties.

Flores et al. [16] used a quadratic BIM algorithm, through a complex valued U-NET, with the database in [13]. Khoshdel et al. [17] performed tumor detection using a 3D, automatically generated random breast tumor database. Marijn Borghouts et al. [18] generated a tumor probability map. Costanzo and Flores used reinforcement learning techniques to perform tumor detection [19].

Our contribution is part of the historical framework of the GeePs-L2S microwave camera [20,21]. We are interested in the possibility of training deep learning models to transform the qualitative images produced by the microwave camera, which represent the distribution of induced currents obtained by backpropagating the measured scattered field into a quantitative image representing the distribution of the object’s dielectric properties. Compared with other techniques, the proposed method uses operations in the spectral domain, making the process less sensitive to noise by taking advantage of high-frequency spatial filtering and less time-consuming with respect to computation. Contributions which associate deep learning with spectral imaging exist in the optical domain [22], but to the best of our knowledge, the use of a spectral approach has not been investigated for quantitative microwave imaging of the breast. In addition, most existing papers on deep learning microwave breast imaging are based on realistic databases created from MRI scans performed on a limited number of patients. Our model is trained on a limited three dimensional database, customized (in-house) from anthropomorphic breast phantoms. There are five major reasons for this investigation: to test the validity of the proposed spectral quantitative method, which works with a limited amount of data; to offer low-cost realistic data that can be easily manipulated; to avoid health and personal protection legislation; to provide anthropomorphic printable models inspired by MRI scans to build up a reference database along with physical phantoms for experimentation; and to easily increase and diversify data, by designing a variety of phantoms of different shapes and sizes. Extension of our 3D database, requiring large-scale production of anthropomorphic cavities and associated simulations, is time consuming. Consequently, this will be investigated in the future. This paper focuses on assessment of the original proposed imaging technique, rather than performance metrics relevant for working with an extensive database. The preliminary results are presented to validate the proposed concept.

This paper is organized as follows: Section 2 highlights the database production process; the anthropomorphic breast model files are used to calculate the truncated spectrum of the induced currents in the planar configuration of the microwave camera. The optimization process including the architecture of the U-NETs and the normalization–denormalization process applied to the data is detailed in Section 2.5. Select numerical results that illustrate both the strengths and limitations of the approach are shown in Section 3. Finally, a discussion about the range of this contribution and its future perspectives is given in Section 4.

## 2. Materials and Methods

### 2.1. Generation of Anthropomorphic Breast Phantoms

**Figure 1:**
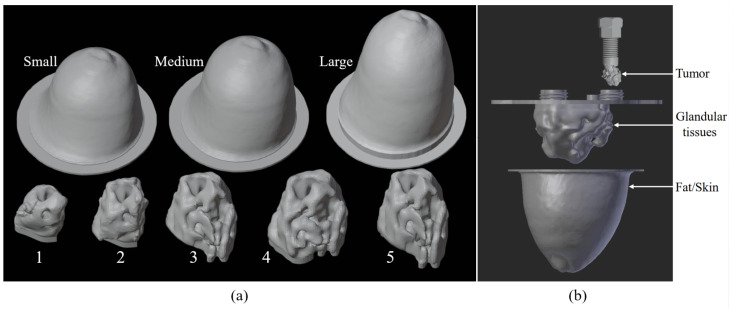
Three-dimensional cavities used to build training database (**a**); example of how to combine different parts of printable phantom (**b**).

To implement deep learning techniques, we created a database based on three-dimensional anthropomorphic models inspired by the GeePs-L2S breast phantom [23,24]. The breast is divided into three different parts—skin-associated fat, glandular tissues, and tumors—as shown in Figure 1. We developed, in addition to the GeePs-L2S breast model, three models for fat and skin and five for glandular tissue. This enabled us to produce 3×5−1=14 models without tumors, as glandular tissue cavity number 5 does not fit inside the smallest fat cavity. This number rose to 14×4=56 without (case 1) or with tumors placed in the adipose (case 2) or glandular (case 3) or both (case 4). In a second step, we considered the mirror of each phantom to double the number of models. Because the phantoms are volumetric, and because of the propagation of the wave, the mirror does not correspond to a 180-degree rotation. Finally, a training set of 56×2×16=1792 images at the center of the models was obtained by rotating them, considering a full rotation corresponding to 16 different views.

**Figure 2:**
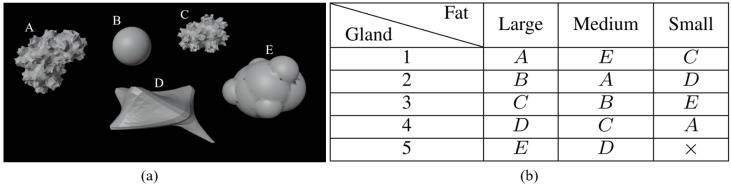
Tumor dataset (**a**); repartition of tumors among cavities (**b**).

Different models of tumors are shown in Figure 2a. Their size varies from 0.5 cm to 2 cm and they are located at different places to create various configurations (cases 2, 3, and 4). Figure 2b, shows the distribution of tumor shapes associated with a letter—A, B, C, D, or E—per cavity. The distribution of tumors is chosen so that each tumor is associated with a cavity only once. As a result, there is no correlation between the use of a specific shape in the phantom and the presence of a tumor in the constructed breast.

However, the training and validation sets are nevertheless correlated, as the current dataset is built from a combination of the various cavities. The data are randomly shuffled and separated, with 80% for training—1433 spectra— and 20% for validation—359 spectra. We set the uncorrelated GeePs-L2S phantom aside for testing. Therefore, the testing set contains 32 spectra (16 rotations and their mirror images). The presented example is used to show that a generalization seems feasible with a wider dataset.

The dielectric properties of the various parts of the phantom are those provided by standard reference databases, e.g., the IFAC [25] or SUPELEC RECIPES [26]. The values of the relative permittivity, $\epsilon_{r}$, and conductivity, σ[S/m], for each tissue used to perform the simulations are given at 2.45GHz in Table 1. Because variability exists for human tissues, we added a ±5% variation value on the dielectric properties as a first try. Higher variabilities will be investigated in the future, as studies in the field mention stronger variations of ±10% due to the dehydration of the tissues [27] or based on canine stroke measurements [28,29]. The values are constant within each cavity due to the simulation constraints (the numerical model is based on a method of moments solution to the surface integral equation). The distribution of permittivity variations between the phantoms is uniform.

### 2.2. Experimental Set-Up: GeePs-L2S Planar Camera

**Figure 3:**
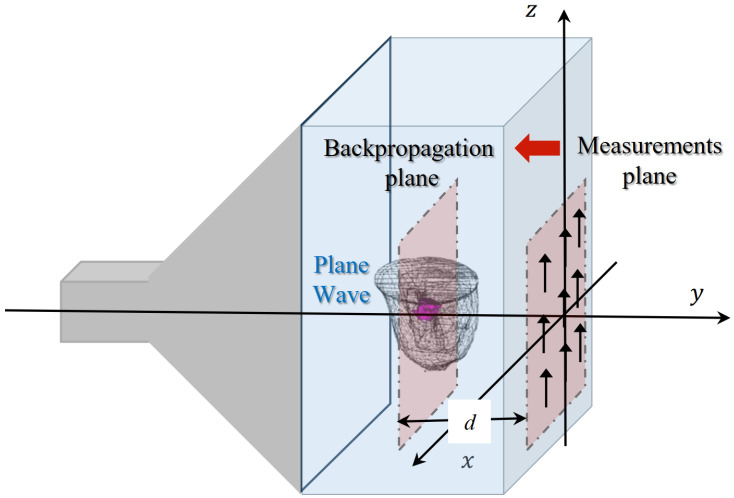
Configuration of GeePs-L2S microwave camera.

The various breast phantoms in the database are studied by placing them inside the GeePs-L2S microwave planar camera as illustrated in Figure 3 [21]. The phantom model is placed in a water tank whose dielectric properties are $\epsilon_{r}=73$ and σ=1[S/m]. The tank is illuminated by a monochromatic plane wave polarized along the z-axis at frequency f=2.45GHz. The scattered field is computed on 64×64 points of the retina (representing the location of the dipoles) using the commercial electromagnetic software WIPL-D [30], based on a surface integral formulation solved by method of moments. This constitutes the simulated data for the imaging problem, solved with a backpropagation algorithm. The observation is placed at d=10cm from the retina, corresponding to the location of a tumor, inside the fatty region of the GeePs-L2S testing phantom, shown in pink in Figure 3. This location choice helps us in investigating other three-dimensional algorithms such as diffraction tomography or deep learning models for volumetric reconstruction. We then compute the filtered spectrum of the induced currents at the tumor position using the Fourier diffraction theorem.

### 2.3. Backpropagation Projection Algorithm

The forward electromagnetic problem states that the induced currents $\underline{j}$ can be computed by solving the integral equation given in (1), where *G* is Green’s function; $\underline{E_{t}}$ is the total electric field; k(x,y,z) is the wavenumber at the point (x,y,z), $k_{water}$ is the wavenumber of the coupling medium (water), ∇ is the operator Nabla, and $\underline{\underline{I}}$ is the identity matrix. A single bar below quantities indicates that they are considered a vector and a double bar stands for matrices.

(1) $$\begin{array}{cc} \underline{E_{t}}\left(x,y,z\right) & =\underline{E_{i}}\left(x,y,z\right)+\left[\underline{\underline{I}}+\frac{\underline{\nabla }.\underline{\nabla }}{k_{water}^{2}}\right].{\;\int \int \int \;}_{V}\;\underline{j}\left(x^{\prime },y^{\prime },z^{\prime }\right)G\left(x,y,z;x^{\prime },y^{\prime },z^{\prime }\right)\;dx^{\prime }\;dy^{\prime }\;dz^{\prime } \end{array}$$

(2) $$\begin{array}{cc} \underline{j}\left(x,y,z\right) & =\left[k^{2}\left(x,y,z\right)-k_{water}^{2}\right]\underline{E_{t}}\left(x,y,z\right) \end{array}$$

The dipoles placed on the retina of the camera measure the vertical component of the total electric field. We deduce the field scattered by the phantom $E_{s,z}$ by subtracting the incident field along the *z*-axis. Assuming that the depolarization inside the phantom is negligible, it is known that Equation (1) can be reduced to a scalar equation. In that way, the scattered field along the z-component on the retina, $E_{s,z}$ at a distance *d* from the object, is then written as follows:

(3) $$\begin{array}{cc} \; & E_{s,z}\left(x,d,z\right)={\;\int \int \int \;}_{V}\;j_{z}\left(x^{\prime },y^{\prime },z^{\prime }\right)G\left(x,d,z;x^{\prime },y^{\prime },z^{\prime }\right)\;dx^{\prime }\;dy^{\prime }\;dz^{\prime } \end{array}$$

Because of Weyl’s angular spectrum expansion, Green’s function can be expressed as follows:

(4) $$\begin{array}{cc} G(x,d,z;x^{\prime },y^{\prime },z^{\prime }) & =-{\;\int \int \;}_{-\infty }^{+\infty }\;\frac{i}{8\pi^{2}\gamma }e^{-i(k_{x}\left(x-x^{\prime }\right)+\gamma |d-y^{\prime }|+k_{z}\left(z-z^{\prime }\right))}dk_{x}\;dk_{z} \end{array}$$

(5) $$\begin{array}{cc} \gamma & =\sqrt{k_{water}^{2}-k_{x}^{2}-k_{z}^{2}} \end{array}$$

If we only consider the propagative waves, Equation (5) enforces that γ∈R. This condition can only be verified in the so-called visible range, a region bounded by a circle of radius $k_{water}=438\;m^{-1}$, according to

(6) $$\begin{array}{c} k_{x}^{2}+k_{z}^{2}\leq k_{water}^{2} \end{array}$$

Some supplementary calculations lead to the Fourier diffraction theorem (7) [31,32]. It stipulates that the Fourier transform of the induced currents ${\hat{j}}_{z}$ is linked to the filtered spectrum, limited to the visible domain, of the scattered field ${\hat{E}}_{s,z}$.

(7) $$\begin{array}{cc} {\hat{E}}_{s,z}\left(k_{x},k_{z}\right) & =-\frac{i}{2\gamma }{\hat{j}}_{z}\left(k_{x},\gamma -k_{water},k_{z}\right)e^{-i\gamma d} \end{array}$$

Then, we can easily deduce the backpropagation projection equation given in (8) [20], which gives an approximation of the induced currents inside the phantom.

(8) $$\begin{array}{c} j_{z}\left(x,z\right)=FT_{2D}^{-1}\left[2i\gamma {\hat{E}}_{s,z}\left(k_{x},k_{z}\right)e^{i\gamma d}\right] \end{array}$$

**Figure 4:**
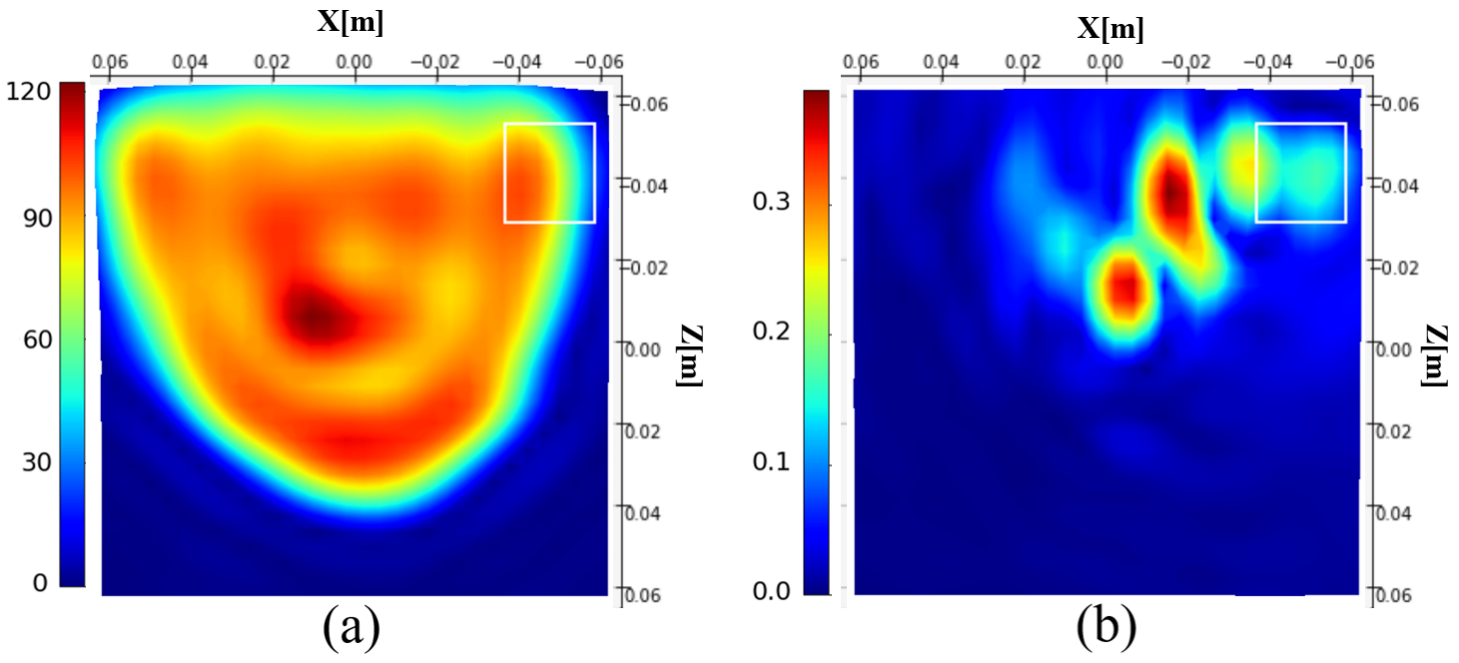
Magnitude of the reconstructed induced current distribution (**a**); differential image (**b**). The white rectangle shows the tumor location.

This formula integrates the induced currents of the entire 3D phantom into a single projective plane, making $|j_{z}|$ given in Figure 4a difficult to interpret. It also means that the projective plane includes information from the different parts of the phantom, making the tumor localization difficult. For assessing the possibility of tumor detection, we calculate the difference between the induced currents with and without the tumor Figure 4b. It appears that tumor information is present in the differential image, demonstrating the sensitivity of the method to the presence of the tumor.

### 2.4. Spectral Filtering

Once the distribution of the scattered field on the retina of the camera has been obtained, it is possible to deduce its spectrum using fast Fourier transform, followed by the spectrum of its currents using (7). Let us introduce the Normalized Spectral Density of ${\hat{j}}_{z}$, noted NSD, given by the following:

(9) $$\begin{array}{c} NSD=10\;log_{10}{\left(\frac{|{\hat{j}}_{z}\left(k_{x},\gamma -k_{water},k_{z}\right)|}{max|{\hat{j}}_{z}\left(k_{x},\gamma -k_{water},k_{z}\right)|}\right)}^{2} \end{array}$$

The measurement is conducted in water with 64 × 64 dipoles spaced $\frac{\lambda_{water}}{4}$. The spectrum ${\hat{E}}_{s,z}$ is therefore composed of 4096 pixels, and its extent is $1751\;m^{-1}$. The Fourier diffraction theorem naturally filters out the high spatial frequencies of the current spectrum ${\hat{j}}_{z}$. The range of the spectrum, according to Equation (6), is bounded by a circle of diameter $876\;m^{-1}$, referred to as the visible range, which reduces the number of pixels to 805 pixels.

**Figure 5:**
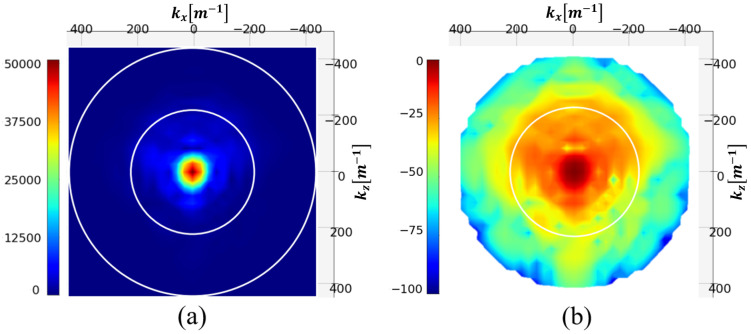
Spectrum (**a**) and NSD [dB] (**b**) of the induced currents. The white circles represent the visible and half-visible domains.

In Figure 5, the modulus of the ${\hat{j}}_{z}$ distribution of the test phantom and its NSD in decibel [dB] are represented. The two white circles represent the limit of the visible range (outer circle) and this same limit divided by two.

The figure shows that the spectrum is almost evanescent outside the visible half-domain. Filtering spatial frequencies outside this circle should therefore not significantly degrade the image. This operation reduces the spectrum to 197 pixels.

Hence, spectral filtering offers several advantages. Firstly, spectrum truncation reduces the number of unknowns to be determined, thereby reducing the complexity of the problem posed, from 4096 to 197 pixels. Secondly, filtering the invisible spectrum and beyond removes the information contained in evanescent waves, which are not accessible due to measurement noises. This natural truncation in the back-propagation algorithm can be assimilated to a regularization technique, making the spectral technique less sensitive to noise addition.

**Figure 6:**
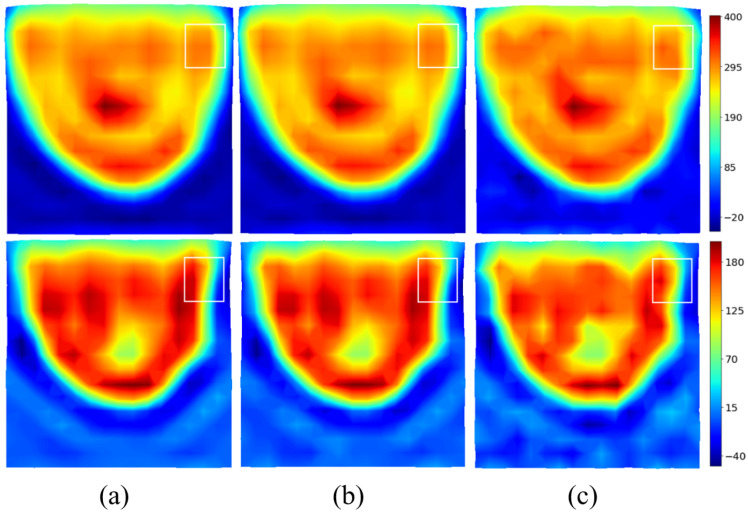
Effect of noise on filtered $j_{z}$ for various SNRs. Top: real part. Bottom: imaginary part. Noiseless (**a**); $SNR_{20\mathrm{dB}}$ (**b**); $SNR_{10\mathrm{dB}}$ (**c**).

The real and imaginary parts of the induced current distributions are reconstructed for different signal-to-noise ratios and shown in Figure 6. Additive White Gaussian Noise (AWGN) is added on $j_{z}$ before filtering. As expected, the method is very robust, and a signal-to-noise ratio (SNR) of 10 dB has a limited effect on the reconstruction. The spectral version of the induced currents—in the visible domain divided by two—will be taken in the following as the input of the U-NETs.

### 2.5. From Qualitative to Quantitative Imaging

Deep learning techniques are online optimization processes. To optimize the spectrum of induced currents, a reference must be chosen. The simplest and most logical approach would be to conduct a qualitative-to-qualitative optimization to optimize the approximate induced currents with respect to the theoretical currents calculated using Equation (2). However, from a computational viewpoint, this choice is not satisfactory. Considering the variations in the field inside the phantom complicates the process of optimizing the induced currents map. The use of the complex contrast *C*, given in Equation (10), seems to be a better choice, as it is strongly linked to different parts of the breast and more challenging.

(10) $$\begin{array}{cc} C(x,z) & =\left(\epsilon^{\prime }\left(x,z\right)-\epsilon_{water}^{\prime }\right)-i\left(\epsilon^{\prime \prime }\left(x,z\right)-\epsilon_{water}^{\prime \prime }\right) \end{array}$$

(11) $$\begin{array}{cc} \epsilon_{water}^{\prime \prime } & =\frac{\sigma_{water}}{\omega \epsilon_{0}} \end{array}$$

In a first step, the data must be standardized and normalized, before training the U-NETs. The choice of scaling method is linked to the values of the parameters to be optimized. Here, the imaginary part of the dielectric contrast and $j_{z}$ currents is small compared to their real part, which can lead to balance problems when the error is backpropagated to the neural network. Min–max normalization ensures that real and imaginary parts follow equivalent dynamics. For this reason, $j_{z}$ and the reference contrast C are rescaled to the interval [0, 1] using the standard min–max scaling formula on the real and imaginary parts.

(12) $$\begin{array}{cc} j_{min-max} & =\frac{ℜ\left(j_{z}\right)-min\left(ℜ\left(j_{z}\right)\right)}{max\left(ℜ\left(j_{z}\right)\right)-min\left(ℜ\left(j_{z}\right)\right)}+i\frac{ℑ\left(j_{z}\right)-min\left(ℑ\left(j_{z}\right)\right)}{max\left(ℑ\left(j_{z}\right)\right)-min\left(ℑ\left(j_{z}\right)\right)} \end{array}$$

(13) $$\begin{array}{cc} C_{min-max} & =\frac{ℜ\left(C\right)-min(ℜ\left(C\right))}{max(ℜ\left(C\right))-min(ℜ\left(C\right))}+i\frac{ℑ\left(C\right)-min(ℑ\left(C\right))}{max(ℑ\left(C\right))-min(ℑ\left(C\right))} \end{array}$$

To avoid the exploding gradient issue during the U-NET backpropagation process and because deep learning models are pseudo-probabilistic models, the input data should be close to 0. It is therefore desirable for the real and imaginary parts of the spectrum to lie within the interval [−1,1]. Hence, a simple normalization is applied to generate the input ${\hat{j}}_{norm}$ and reference spectra ${\hat{C}}_{norm}$ used in the optimization process. The applied operation preserves the Fourier relationship as it only modifies the magnitude and not the phase of the quantities:

(14) $$\begin{array}{cc} {\hat{j}}_{norm}\left(k_{x},k_{z}\right) & =\frac{{\hat{j}}_{min-max}\left(k_{x},k_{z}\right)}{max(|{\hat{j}}_{min-max}\left(k_{x},k_{z}\right)|)} \end{array}$$

(15) $$\begin{array}{cc} {\hat{C}}_{norm}\left(k_{x},k_{z}\right) & =\frac{{\hat{C}}_{min-max}\left(k_{x},k_{z}\right)}{max(|{\hat{C}}_{min-max}\left(k_{x},k_{z}\right)|)} \end{array}$$

**Figure 7:**
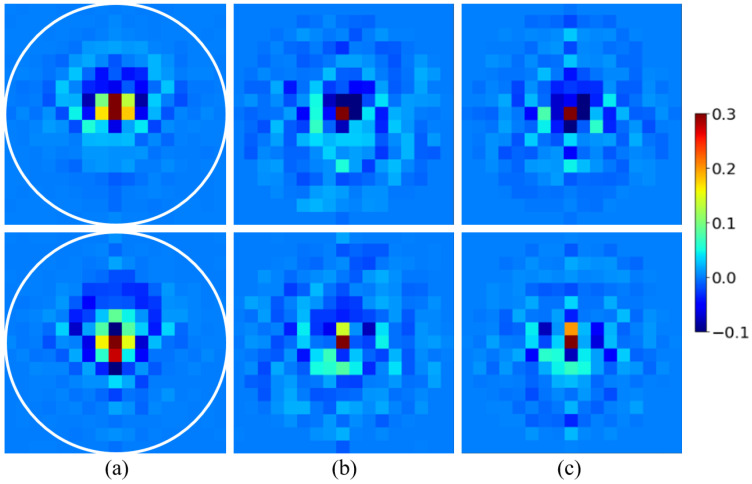
Input, reference, and output of the U-NETs for the testing spectrum. The white circle radius is half the visible domain. Top: real part. Bottom: imaginary part. ${\hat{j}}_{norm}$ (**a**); ${\hat{C}}_{norm}$ (**b**); ${\hat{C}}_{net}$ (**c**).

The aim of the U-NET pair is to perform a full transformation of the input ${\hat{j}}_{norm}$ into the output contrast ${\hat{C}}_{net}$, using ${\hat{C}}_{norm}$ as a reference. The input, reference, and output of the U-NETs for the test example are shown in Figure 7.

In a second step, the denormalization and destandardization process required to compute $\epsilon_{rnet}$ and $\sigma_{net}$ values from the $C_{net}$ contrast reconstructed by the U-NETs is conducted as follows. We introduce $M_{ℜ}$ and $M_{ℑ}$, the mean of the real or imaginary value of all the pixels outside the phantom on the reconstructed image (in water), respectively. As the dielectric properties of water are known, the operations given in (16) and (17) calibrate the reconstructed values, $\epsilon_{cal}$ and $\sigma_{cal}$, with respect to the water, making the process less sensitive to oscillations caused by spectral reconstruction errors.

(16) $$\begin{array}{cc} \epsilon_{cal}\left(x,z\right) & =\left(\frac{ℜ(C_{net}\left(x,z\right))}{M_{ℜ}}-1\right)\epsilon_{rwater} \end{array}$$

(17) $$\begin{array}{cc} \sigma_{cal}\left(x,z\right) & =\left(\frac{ℑ(C_{net}\left(x,z\right))}{M_{ℑ}}-1\right)\sigma_{water} \end{array}$$

By using min–max scaling a second time, we can rescale $\epsilon_{cal}$ and $\sigma_{cal}$ in the interval $\left[min(|\epsilon_{cal}\left|),max(\right|\epsilon_{cal}\left|\right)\right]$ and $\left[min(|\sigma_{cal}\left|),max(\right|\sigma_{cal}\left|\right)\right]$ to enforce that the maximum and the minimum values reconstructed by the U-NETs are positive. That way, Equations (18) and (19) give us the dielectric properties $\epsilon_{rnet}$ and $\sigma_{net}$ reconstructed by the U-NETs.

(18) $$\begin{array}{cc} \epsilon_{rnet} & =min(|\epsilon_{cal}\left|\right)+\left(max(\right|\epsilon_{cal}\left|\right)-min(|\epsilon_{cal}|)).\left[\frac{\epsilon_{cal}-min\left(\epsilon_{cal}\right)}{max\left(\epsilon_{cal}\right)-min\left(\epsilon_{cal}\right)}\right] \end{array}$$

(19) $$\begin{array}{cc} \sigma_{net} & =max(|\sigma_{cal}\left|\right)+\left(min(\right|\sigma_{cal}\left|\right)-max(|\sigma_{cal}|)).\left[\frac{\sigma_{cal}-min\left(\sigma_{cal}\right)}{max\left(\sigma_{cal}\right)-min\left(\sigma_{cal}\right)}\right] \end{array}$$

Finally, the application of the contour adds prior information by enforcing the constant values of $\epsilon_{rwater}$ and $\sigma_{water}$ outside the phantom.

### 2.6. U-NETs for Spectral Reconstruction of Dielectric Contrast

**Figure 8:**
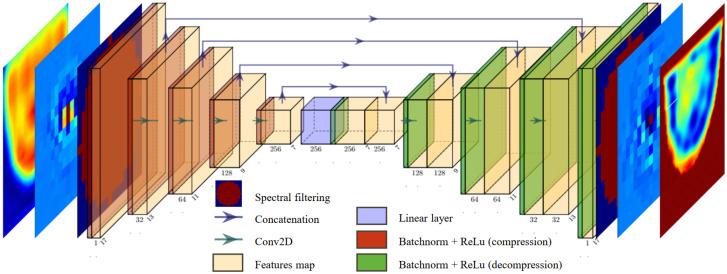
U-NET for image enhancement through spectrum transformation.

The model was fully developed hand-made in Python, using functions from the open-source PyTorch library [33]. For spectrum enhancement, we chose to use a pair of identical U-NETs [34], with the architecture illustrated in Figure 8. One U-NET is designed to optimize the real part of the spectrum ${\hat{C}}_{net}$, while the second U-NET focuses on the imaginary part. The spectral filter is applied both on the spectral input and output of the U-NET pair. That way, the U-NETs exclusively optimizes the filtered part and performs a proper pixel-to-pixel transformation of the spectrum.

To perform the reconstructions, the spectra are grouped into batches of three, containing a total of n=17×17×3 parameters. In the compression phase, we implement a series of filters with decreasing sizes, using zero-padding adjustments to ensure gradual compression of the spectrum without employing pooling operations. This allows the information to be compressed through convolutions, reducing the dimensions of each feature map from 17×17 to 7×7. The various applied filters gradually generate an increasing number of feature maps, gathering the statistical information needed to modify the spectrum.

During the decompression phase, the filter dimensions are unchanged, and the padding is gradually increased to the initial spectrum size at the end of the network. The features of the U-NETs architecture are presented in Table 2.

Before each convolution, batch normalization is applied to speed up the learning process, and the ReLu function is introduced to add non-linearity to the U-NETs. The transition from compression to decompression is a linear layer.

The Adam optimizer [35] minimizes the Weighted Mean Absolute Percentage Error (WMAPE) loss for the real and imaginary components of the spectrum, given by Equations (20) and (21), respectively. The optimizer parameters are set to their default values: learning rate (lr)=0.001 and inertial coefficients (β1,β2)=(0.9,0.999).

(20) $$\begin{array}{cc} loss_{ℜ} & =\frac{\sum_{i=1}^{n}\left|ℜ\left({\hat{C}}_{net}\right)-ℜ\left({\hat{C}}_{norm}\right)\right|}{\sum_{i=1}^{n}\left|ℜ\left({\hat{C}}_{norm}\right)\right|} \end{array}$$

(21) $$\begin{array}{cc} loss_{ℑ} & =\frac{\sum_{i=1}^{n}\left|ℑ\left({\hat{C}}_{net}\right)-ℑ\left({\hat{C}}_{norm}\right)\right|}{\sum_{i=1}^{n}\left|ℑ\left({\hat{C}}_{norm}\right)\right|} \end{array}$$

**Figure 9:**
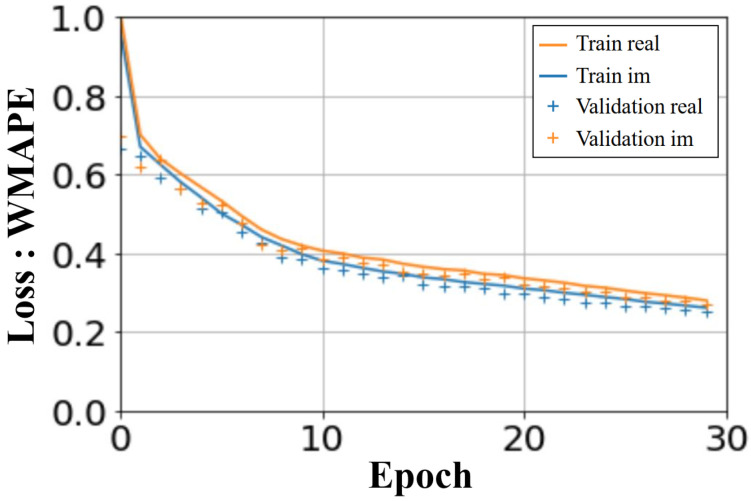
U-NETs: WMAPE loss on validation and training sets versus number of epochs.

The U-NETs were trained over 30 epochs. The computation time for the learning phase was less than an hour and a half, on an NVIDIA RTX A2000 8GB. The computed loss in Figure 9 shows the WMAPE loss function as a function of the number of epochs. The loss values displayed are average values, taking into account all the batches processed. A similar convergence is observed for the learning and validation process, for both real and imaginary parts.

## 3. Results

### 3.1. Validation Results

The validation set of 359 spectra contains 20% of the data. The U-NETs were trained with noise-free data in order to assess their effectiveness on well-controlled data.

**Figure 10:**
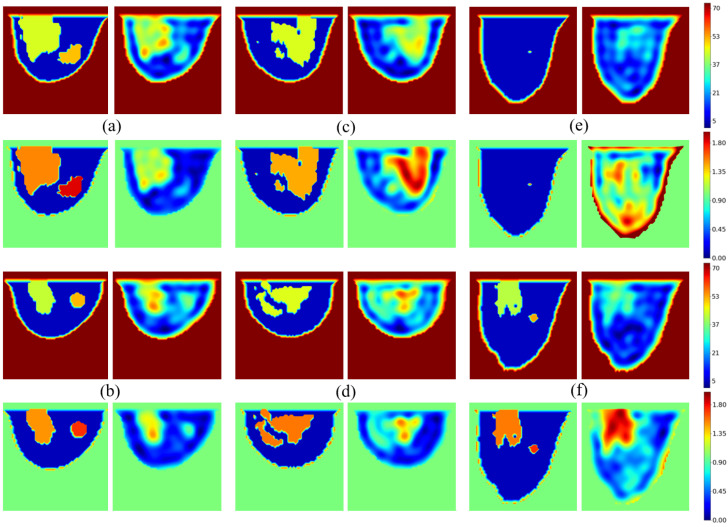
Validation examples: phantoms with tumor (**a**,**b**); phantoms without tumor (**c**,**d**); failure cases (**e**,**f**). Color scale is fixed to [1,73] on $\epsilon_{r}$ and [0,2][S/m] on σ to fit all values distant of ±5% from standard ones.

As the models are built from various combinations of fat, skin, glandular tissues, and tumors, the validation dataset is correlated with the training dataset, which generates overfitting issues in our model. It should also be noted that, since the database models are three-dimensional, tumors are not necessarily present in the central plane of observation. The results are shown in Figure 10. Six models are considered, including three with tumors in the central slice. Four images are presented for each model; the first column corresponds to the exact profile of the dielectric constant (top) and conductivity (bottom), and the second column gives the corresponding profiles reconstructed by the U-NETs.

The network generally produces a high-quality image of permittivity and conductivity distributions. The shapes of the various tissues—skin, fat, and glandular—are well reproduced, with values close to the reference ones. The result is more questionable when the model contains tumors, or more generally for “unusual” situations, leading to failure cases. In the examples shown in Figure 10, we have intentionally selected failure situations. As we can see, the tumor appears in the right place in tumor Models Figure 10a,b, but not in Model Figure 10f. The reason is probably linked to the training dataset, since 90% of it is generated from tumor-free breast models in the central breast slice. Similarly, as shown in Figure 10e, the neural network faces issues when reconstructing glandular-free models, creating artifacts inside the breast, as only 5% of the generated slices are devoid of glandular tissues.

**Figure 11:**
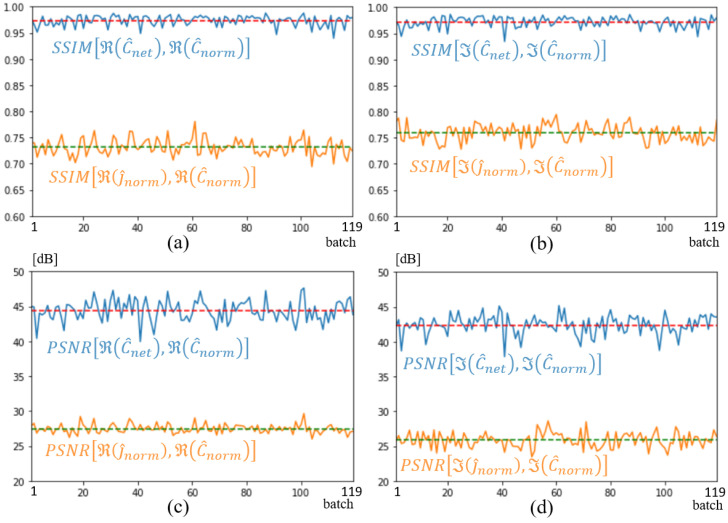
SSIM (**a**,**b**) and PSNR (**c**,**d**) metrics on ${\hat{j}}_{norm}$ input, shown in orange, and ${\hat{C}}_{net}$ output shown in blue. Dotted lines represent average metric over validation dataset. Real part (**a**,**c**); imaginary part (**b**,**d**).

Figure 11 shows the structural similarity (SSIM) [36,37] and the peak signal-to-noise ratio (PSNR) that [37] computed with torchmetrics default parameters, over the entire validation dataset of 359 spectra. As simulations are run in batches of 3, the metrics are plotted versus the 119 batches. The yellow and blue curves correspond to an evaluation of image quality at the input and output of the U-NETs, respectively. As can be seen, the overall improvement brought about by the U-NETs is major, with an average SSIM of 0.97 and a gain of over 20 dB according to the PSNR metric, and on both the real and imaginary parts of the spectrum. In comparison with Figure 11d,c, the imaginary part of the spectrum appears slightly less well optimized than the real part of the spectrum.

To further improve these results, the training and validation datasets need to be expanded. This can be achieved easily and significantly by adding tumors in the center and diversifying the geometric shapes of the breast models, as this will have a large impact on the total number of possible combinations. Also, with the enlargement of the database, a better decorrelation between the training and the testing data could be achieved. These results, obtained with a limited database and a qualitative input image of very low resolution, are very encouraging as preliminary results.

### 3.2. Test Results

**Figure 12:**
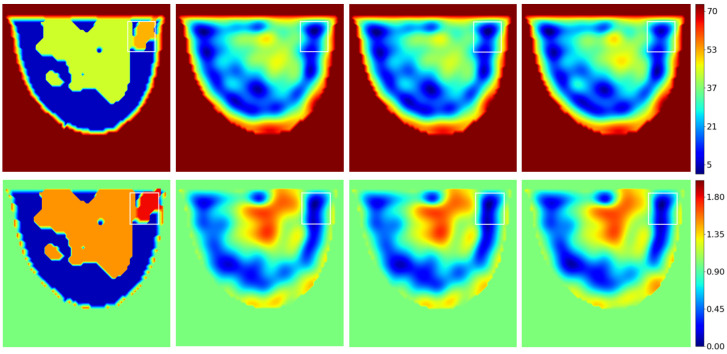
Test example. **Top**: $\epsilon_{r}$. **Bottom**: σ[S/m]. From **left** to **right**: reference; noiseless reconstruction; SNR 20 dB reconstruction; SNR 10 dB reconstruction. The white rectangle shows the tumor location.

For now, the testing set is limited to the 32 slices of the GeePs-L2S breast phantom. Figure 12 shows the dielectric constant and conductivity profiles provided by the network for the slice passing through the tumor and for different signal-to-noise ratios. The effect of noise is almost negligible, confirming the method’s low sensitivity to noise for the reasons outlined in Section 3.

An improvement can be seen both in terms of spatial resolution and of what the image represents. The U-NET improves spectrum quality by transforming the low-spatial-resolution qualitative image provided by the backpropagation algorithm into a higher-spatial-resolution quantitative image, providing access to the phantom’s dielectric properties, but not those of the tumor.

**Figure 13:**
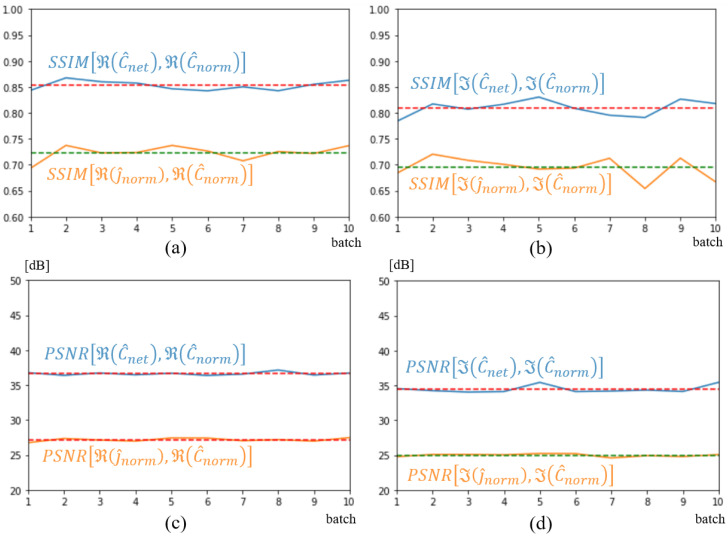
SSIM (**a**,**b**) and PSNR (**c**,**d**) metrics on ${\hat{j}}_{norm}$ input, shown in orange, and ${\hat{C}}_{net}$ output shown in blue. Dotted lines represent average metric over test dataset. Real part (**a**,**c**); imaginary part (**b**,**d**).

As before, SSIM and PSNR values were calculated on the 32 spectra of the test database and depicted in Figure 13. As expected, given the size of the database, the network output (blue curves) shows a lower SSIM than in the previous case, a value between 0.8 and 0.85. This result aligns with the lower-quality image reconstruction depicted in Figure 12 This reflects the neural network’s difficulty in reconstructing a novel tumor in a new location. However, if we compare these values with those obtained with the network’s input quantities (yellow curves in the figures), the improvement remains significant for both real and imaginary parts. U-NETs bring an average improvement of 10 dB on the PSNR of the reconstructed spectrum.

## 4. Discussion

The main objective of this paper was to develop a combination of U-NETs with a spectral method to produce real-time quantitative breast imaging. The preliminary results presented in the previous section validate the method and confirm its potential in terms of efficiency with an SSIM of 0.97 in validation and 0.83 in testing and robustness to a low SNR of 10 dB. A second achievement was the development of an in-house, realistic, diverse, low-cost, and easy-to-use 3D database. To our knowledge, both developments are new.

However, this work is a first step. Indeed, as research on model architectures such as CNN, U-NET, ANN, and CVCNN remain important, the most essential aspect of deep learning remains the quality and quantity of the database and data pre/post-processing, as the neural network parameters are optimized from the available dataset. Unfortunately, the number of breast phantoms currently available worldwide remains limited. In the literature, studies that clearly avoid data leakage by using uncorrelated testing and training sets, achieving deep learning generalization results during image enhancement, dispose either of a large amount of automatically generated data [15] or of information from other modalities [12]. This is not the case here. Consequently, overfitting is mandatory with such a small database, and the correlation between training and validation sets is ensured by the combinatorial nature of the training/validation set. This is clearly an obstacle to generalization, and techniques such as regularization or dropout are useless when facing a lack of data.

To solve this issue, we propose in this study to begin with a small in-house database, with the idea of optimizing and extending it in the future. The idea is to study both the reconstruction process and the data themselves in an optimized way. This would enable us to better understand the behavior of the neural network as a function of the database, i.e., its size, processing, and potential biases. The database would become a customizable input, and the optimization process would be seen as a unified optimization of the model and data.

This database must be realistic, diversified, and easy to develop. This is why we propose a combination of anthropomorphic breast cavities, derived from modifications made to a realistic phantom, generated from an MRI scanner [13]. Working with phantoms not only frees us from legal and ethical issues, but also allows us to control the entire database development program. Moreover, the existence of printable twins of constructed cavities can be used for experiments or to calibrate measurements.

However, conducting research in this way comes at a cost both in terms of development time and effort. This is why this study focuses primarily on assessing the validity of the method rather than its performance.

Does the combination of U-NETs with a spectral method of very low computational cost, compared to classical optimization methods—DBIM, CSI, GN, etc.—yield promising results ? The answer is positive, if we refer to the images and associated metrics presented in the previous section.

## 5. Conclusions

The deep learning approach implemented in this paper seems well suited to the complex problem of retrieving the dielectric properties of an anthropomorphic breast model from the spectrum of induced currents approximated by a sufficiently well-trained neural network. Preliminary results are impressive, with significant improvements in both image contrast and spatial resolution. However, this efficiency would be even more effective with a well-dimensioned database whose development considers the behavior of the neural network in the face of statistical biases and correlations that the database may introduce. Although this database is not currently available, the choice of working in the spectral domain with the backpropagation algorithm seems attractive. The proposed method is a real-time process, robust to noise, and easy to implement for the purpose of evaluating the effectiveness of deep learning. It is potentially a good candidate for future imaging systems.

## Figures and Tables

**Table 1:** $\epsilon_{r}$ and σ for the four tissues of the phantom.

	Fat	Glandular	Skin (Wet)	Tumor
$\epsilon_{r}\left(\pm 5\%\right)$	5	44	42	53
σ(±5%) [S/m]	0.1	1.5	1.6	1.8

**Table 2:** Features of U-NETs.

	Compression				Linear Layer	Decompression			
Step	1	2	3	4	5	6	7	8	9
Feature map size	13×13	11×11	9×9	7×7	7×7	7×7	9×9	11×11	13×13
Feature map number	32	64	128	256	-	256+256	128+128	64+64	32+32
Filter size	9×9	7×7	5×5	3×3	-	3×3	3×3	3×3	3×3
Padding	2	2	1	0	-	2	2	2	3

## Data Availability

The original contributions presented in this study are included in the article. Further inquiries can be directed to the corresponding author.
